# An American Commission for the Study of Tropical Diseases

**Published:** 1899-04-15

**Authors:** 


### AN AMERICAN COMMISSION FOR THE STUDY OF TROPICAL DISEASES.

The Johns Hopkins University has just sent off a scientific expedition to study tropical diseases. The places visited will include Yokohama, Kobe, Shanghai, Hong Kong, Singapore, and Colombo, but most of the actual work will be done at Manilla.

